# Are we cultivating the perfect storm for a human avian influenza pandemic?

**DOI:** 10.1186/s40659-024-00570-6

**Published:** 2024-12-19

**Authors:** Tomas Perez-Acle, Cesar Ravello, Mario Rosemblatt

**Affiliations:** 1 Computational Biology Laboratory, Fundacion Ciencia & Vida, Universidad San Sebastian, Avda. del Valle Norte 725, Huechuraba, 8580702 Santiago, Region Metropolitana Chile; 2 Facultad de Ingeniería, Arquitectura y Diseño, Universidad San Sebastian, Bellavista 7, Recoleta, 8420524 Santiago, Region Metropolitana Chile; 3 Laboratory of Cellular and Molecular Inmunology, Fundacion Ciencia & Vida, Universidad San Sebastian, Avda. del Valle Norte 725, Huechuraba, 8580702 Santiago, Region Metropolitana Chile; 4 Facultad de Medicina y Ciencia, Universidad San Sebastian, Lota 2465, 7510157 Santiago, Region Metropolitana Chile

**Keywords:** H5N1, HPAI, Dairy cattle, Pandemic

## Abstract

The emergence of highly pathogenic avian influenza (HPAI) A H5N1 virus in dairy cattle marks a troubling new chapter in the ongoing battle against zoonotic diseases. Since its initial detection in 1955, the H5N1 virus has primarily been associated with poultry, posing significant threats to both animal and human health. However, recent outbreaks in U.S. dairy herds across nine states have revealed an alarming expansion of the virus, with over 190 herds affected as of September 2024. This unprecedented spread in cattle has sparked intense concern among scientists and health officials, especially with reports indicating that up to 20% of dairy products may contain traces of the virus. The implications of the H5N1 virus establishing itself in cattle populations are profound. This potential endemic presence could transform dairy farms into reservoirs of the virus, facilitating its evolution and increasing the risk of human transmission. Mutations enhancing viral replication in mammals have already been identified, including the notorious PB2 E627K mutation linked to increased virulence. Moreover, the detection of the virus in the central nervous system of infected animals, including cats, underscores the broad tissue tropism and severe pathogenic potential of the H5N1 virus. Current containment efforts include stringent biosecurity measures and financial incentives for enhanced testing and personal protective equipment (PPE) for farmers. Yet, gaps in testing infrastructure and the resurgence of raw milk consumption pose significant challenges. The U.S. Department of Agriculture (USDA) and the Centers for Disease Control and Prevention (CDC) emphasize the critical need for comprehensive testing and pasteurization to mitigate the risk of human infection. As the scientific community races to adapt existing antiviral treatments and develop effective vaccines, the concept of a One Health approach becomes increasingly vital. This holistic strategy calls for coordinated actions across human, animal, and environmental health sectors to preemptively tackle emerging zoonotic threats. Strengthening surveillance, fostering international cooperation, and investing in research are essential steps to prevent the H5N1 virus from igniting the next global health crisis. The current avian influenza outbreak serves as a stark reminder of the delicate balance between human activities and viral evolution. Our collective ability to respond effectively and proactively will determine whether we can avert the perfect storm brewing on the horizon.

## Introduction

### Dairy cattle infection with H5N1 virus: a new endemic disease?

Although the first human case was detected in a child in Hong Kong in 1997 [1], historical records of the disease, initially known as ‘fowl plague’, date back to 1878, being characterized as a severe and rapidly spreading disease affecting chickens [2]. Despite its earlier characterization, the etiological agent remained unknown until 1955, when it was identified as a Type A influenza virus (orthomyxovirus). The term ‘highly pathogenic avian influenza (HPAI)’ came into use until 1981 during the first International Symposium on avian influenza held in 1981 in Beltsville, Maryland, United States of America (U.S.) [2].

Since its first reported outbreak in 1959 (A/chicken/Scotland/59) [3], the HPAI H5N1 variant has emerged as a global threat, rapidly spreading across the world [4, 5]. To date, more than 900 zoonotic human infections have been reported in 23 countries [6]. The case fatality rate (CFR) of the HPAI H5N1 virus in humans is significantly high, ranging from 59% to 66%, underscoring its severe impact on human health [7–9].

**Fig. 1:**
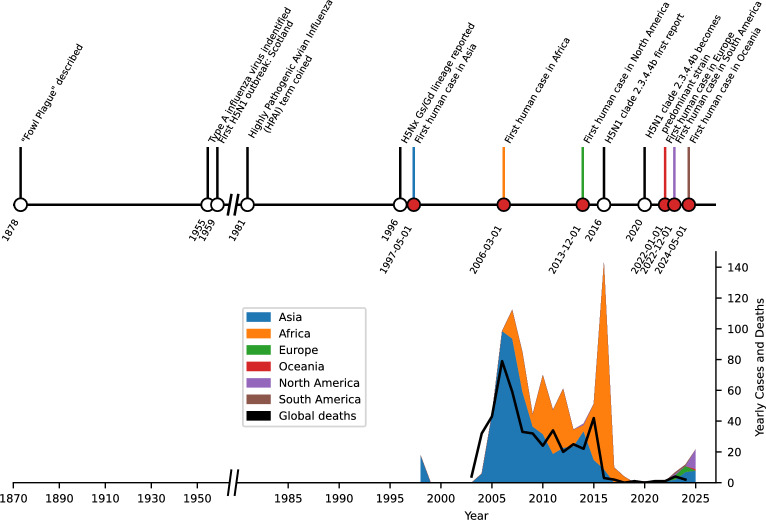
Global timeline of highly pathogenic avian influenza (HPAI) outbreaks and key events. The timeline spans from the late 19th century to the present, highlighting as red dots the first identified human cases of avian influenza in various continents, as well as the evolution of the virus. The graph also tracks the yearly global human cases and deaths associated with H5N1 virus outbreaks. While cases in the current decade are still low, the recent increase in affected people is of concern

Traditionally, the HPAI virus primarily affects birds [10] leading to the destruction of millions of poultry worldwide, significantly impacting food security and livelihoods [11]. However, since 2020, the H5N1 clade 2.3.4.4b [12] emerged as a predominant strain in wild birds and poultry across multiple continents, posing significant threats to both animal and human health due to its widespread dissemination and genetic diversity [13, 14]. These precedents are summarized in Fig. 1.

**Fig. 2:**
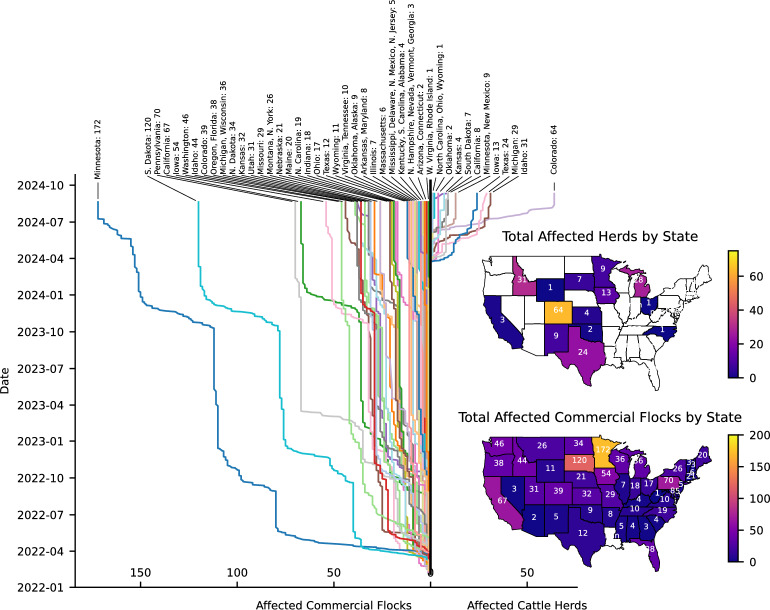
Spatial distribution and temporal progression of affected commercial flocks and cattle herds by state from January 2022 to September 2024. The graph tracks the number of affected herds and flocks across various states, including notable peaks in states such as California, Iowa, Minnesota, and South Dakota. The data highlights the variability of outbreaks over time, with different states showing varying levels of impact. The color-coded bars represent the cummulative number of affected commercial flocks and cattle herds, for each state illustrating the widespread nature of the outbreaks across the United States. Even though spread in herds has begun only recently, the propagation has been faster than for flocks. The spatial distribution of affected farms is very different depending on the type of animals

Recent reports of HPAI H5N1 virus infecting dairy cattle in the U.S. have sparked significant concern [15]. As shown in Fig. 2, as of September 2024, detections in over 190 dairy herds in 14 states-Texas, Kansas, Michigan, New Mexico, Idaho, Ohio, North Carolina, South Dakota, Colorado, Minnesota, Wyoming, Iowa, Oklahoma and California-based on the confirmation date, indicate widespread dispersion across the U.S. [16].

The U.S. Department of Agriculture (USDA) and the Centers for Disease Control and Prevention (CDC) have recommended the implementation of enhanced biosecurity measures to contain the outbreak [16]. These measures include restricting farm access, segregating infected animals, and ensuring that milk from sick cows does not enter the food supply [17]. Moreover, the USDA has recently introduced financial incentives to encourage testing and the use of personal protection elements (PPE) among farmers. These incentives include funding to enhance biosecurity measures and cover costs related to additional testing and PPE for employees [18].

In spite of these containment efforts, recent studies have revealed a concerning prevalence of H5N1 virus RNA in dairy products, with findings indicating that approximately one in five dairy products may contain remnants of the virus [19]. Further studies have shown that pasteurization effectively inactivates the H5N1 virus, leading to the U.S. Food and Drug Administration (FDA) to confirm that pasteurized milk and dairy products, including cheese, are safe for consumption [20]. Unexpectedly, a revival of raw milk consumption, particularly that coming from herds infected with H5N1 virus, has surged in the U.S. [21, 22]. Although no human cases have been directly linked to drinking raw milk, the FDA strongly advises against the consumption of raw milk and raw milk derived products from infected cows due to the presence of potentially harmful pathogens, including the H5N1 virus [20, 23]. This recommendation is supported by a recent study in which oral inoculation of raw milk from herds infected with the HPAI H5N1 virus caused systemic infections in mice [24].

Of note, while in the midst of these unprecedented massive outbreaks detected in more than 190 herds in the U.S., as of September 2024 (Fig. 2), significant gaps in the readiness to detect and manage avian influenza outbreaks still remain [25]. The scarcity of tests, which hinders timely identification of the virus, particularly in farmworker communities, and the role of health insurance constraints and FDA regulations in limiting testing availability are all components hindering a more accurate situational awareness. The current testing infrastructure seems insufficient to comprehensively monitor and control the spread of HPAI H5N1 virus [26]. While the USDA has mandated testing for interstate movement of dairy cattle, this measure does not extend to all herds or consistently include the totality of farm workers who may be exposed to the virus, nor does it include non-symptomatic dairy cows [27].

In a recent effort to overcome these limitations, the CDC is seeking to expand testing capacity for H5N1 virus in people by addressing current diagnostic infrastructure shortcomings and the challenges posed by regulatory requirements and funding constraints. The CDC’s initiative aims to surmount these obstacles by increasing testing availability and enhancing outreach to high-risk populations, ensuring a more robust defense against potential avian influenza outbreaks in humans [28].

Since February 2022, the CDC, along with state and local health departments, has been monitoring individuals exposed to infected birds, poultry, or other animals for a 10-day period post-exposure. Over this span, more than 10,000 people have been monitored and up to 370 individuals have been tested for the HPAI H5N1 virus. In response to the ongoing HPAI outbreak in cattle, similar monitoring efforts have been applied to those exposed to infected cattle. From March 2024 to the present, at least 1300 individuals have been monitored. As of July 2024, 61 of these individuals have been tested for HPAI H5N1 virus [29].

The widespread of herds infection together with the presence of HPAI H5N1 virus RNA in dairy products, suggest that the infection in herds could be much larger than previously thought [30]. Thus, the possibility that the H5N1 virus could become endemic in dairy cattle in the U.S. is a growing concern [31, 32]. If the virus establishes itself in cattle populations, it could persist and circulate within herds, making it more challenging to eradicate. This scenario could lead to continuous low-level infections and sporadic outbreaks, or even worst, to create in vivo laboratories allowing the virus to evolve in mammals which are in very close contact to humans.

## Viral evolution in real time: back to the Spanish flu

The H5N1 virus, which has been affecting dairy cattle and, as of July 2024, has infected four farm workers in the U.S. [33]-one from Texas, two from Michigan and one from Colorado-exhibits mutations that could potentially increase the risk of human-to-human transmission [34, 35]. A key mutation identified in the virus from the first case from Texas, A/Texas/37/2024, which is also present in one sequence report from cattle [36] is PB2 E627K [37]. This mutation is known to enhance the virus’s ability to replicate efficiently in mammalian hosts [38]. Of note, PB2 E627K has been previously observed in other mammals infected with the H5N1 virus, indicating a significant adaptation to mammalian cells [39, 40].

The sequences from the H5N1 virus infecting the second human case, the first reported in Michigan, A/Michigan/90/2024, lacked the PB2 E627K mutation found in the Texas case but contained the PB2 M631L mutation, which is associated with viral adaptation in mammalian hosts. This mutation is present in 99% of dairy cow sequences but is only sporadically observed in birds [36, 41].

Fortunately, in both cases, the infection remained mild, characterized mainly by conjunctivitis, and responded well to antiviral treatment with oseltamivir [37].

Of note, the third human case, also from Michigan, exhibited more typical symptoms of acute respiratory illness associated with influenza virus infection. Among others, the patient reported upper respiratory tract symptoms including cough without fever and eye discomfort with watery discharge [42]. Despite the patient recovered after treatment with oseltamivir, the exhibition of upper respiratory tract symptoms is of concern due to the potential for virus spread via aerosols through coughing.

Another critical mutation present in the H5N1 virus is PB2 T271A, which has been reported in infected minks in Spain [43]. This mutation is particularly important because it was also present in the H1N1 virus responsible for the 2009 pandemic [44], suggesting it plays a role in enhancing the virus’s transmissibility among mammals. The presence of this mutation in various mammal species infected with the H1N1 virus, especially those like minks and pigs that can act as mixing vessels for different influenza viruses, is of high concern [45, 46]. In the case of co-infection between H5N1, H1N1, and other influenza A variants in these animals, new viral strains capable of efficient human-to-human transmission could emerge through genetic reassortment events [47].

On March 29, 2023, Chile reported its first human infection of HPAI H5N1 virus, marking the second such case in South America after a January 2023 case in Ecuador [48, 49]. The PB2 D701N mutation was identified following the isolation and sequencing of the virus. This mutation, located in the C-terminal domain of PB2, is linked to increased virulence and transmissibility in mammals, as demonstrated by experiments in mice, guinea pigs, and ferrets [50–53]. It has also been previously found in human H5N1 HPAI virus infections in Asia, with no evidence of human-to-human transmission [54, 55].

Significantly, the PB2 D701N mutation, along with other amino acid substitutions (Q591K in the PB2 gene, R57Q in the PA gene, and V226T in the NS gene), was detected during recent outbreaks among sea lions in Brazil, Chile, and Peru [56, 57]. The widespread presence of this mutation in both terrestrial and aquatic mammals, including red foxes, lynx, black bears, and seals [58], and more recently in dairy cattle [36], underscores its role in mammalian adaptability and pathogenicity.

Other significant mutations observed in the HPAI H5N1 virus infecting dairy cattle include a set of substitutions in the HA protein, such as 137A, 158N, and 160A (using H3 influenza subtype number) [59]. Denoting the ongoing viral adaptation occurring in cattle, these mutations have been documented to increase the affinity of avian influenza viruses for human-type receptors [60, 61].

Surprisingly, it has been found that both avian and mammalian receptors are present in the mammary glands of dairy cows [62]. This dual presence of receptors can facilitate the binding and replication of the virus in a way that promotes cross-species transmission.

The implication of these findings is profound. The potential for the virus to become endemic and the presence of multi-species receptors raises significant concerns about infected cattle becoming reservoirs for the HPAI H5N1 virus, facilitating human contagion [32].

During the 1918–1919 Spanish influenza pandemic, pigs may have played a notable role in the epidemiology of the disease, as the virus was a novel H1N1 strain with genetic material from avian and swine influenza viruses [63]. A similar phenomenon ocurred in Mexico in 2009, producing the 2009 swine flu pandemic [64]. Pigs, known as mixing vessels, can be infected by both avian and human influenza viruses, facilitating the reassortment and emergence of new influenza strains [47, 65].

As of June 2024, dairy cows, another mammal which lives in close contact with humans, may play a similar role to that of pigs during the Spanish flu pandemic.

## More than a flu: a viral infection of the central nervous system

Recent cases of H5N1 virus infections in cats on dairy farms in the U.S. have raised significant concerns due to the Central Nervous System (CNS) tropism of the HPAI virus [66]. Post-mortem analyses of the cats exhibited severe systemic viral infections with notable CNS damage. This included severe subacute multifocal necrotizing and lymphocytic meningoencephalitis, vasculitis, and neuronal necrosis. Immunohistochemistry revealed positive influenza A virus antigen in brain tissues, particularly in neurons and retinal layers [66]. These findings highlight the significant CNS tropism of the H5N1 virus, demonstrating its ability to replicate in a wider array of mammalian tissues [67].

Evidence from other studies highlight the CNS tropism of the H5N1 virus. Infections in mammals such as mice, ferrets, and wild foxes have shown that the virus can invade and replicate in the CNS, causing neuroinflammation and neurodegeneration [68–71]. Viral antigens and RNA have been found in brain tissues, indicating that the H5N1 virus can cross the blood-brain barrier and infect neural cells [72].

The neurotropism of the H5N1 virus is influenced by a variety of mutations across different genes. Key mutations include those in the NS1 gene (F103L and M106I), PB2 (E158G and M631L), NA (K110E), and NP (K470R) [73–76]. These mutations enhance the virus’s ability to infect and replicate in mammalian neural tissues, contributing to its neurovirulence and neurotoxicity.

Thus, in the eventuality of the HPAI H5N1 virus becoming a human pandemic, its burden could be exacerbated by generating unknown cognitive effects at the population level, leading us into uncharted territory.

## Adapting to change: overcoming H5N1 antiviral resistance and vaccine challenges

The availability of effective treatments and vaccines is crucial in combating a potential HPAI H5N1 pandemic in humans. Antiviral drugs such as oseltamivir (Tamiflu^®^) and zanamivir (Relenza^®^) are currently the primary treatments for H5N1 virus infections and as a profilactic measure for people in close contact with the infected person [37, 77]. These NA (neuraminidase) inhibitors work by preventing the virus from exiting the cell and spreading within the body [78]. However, the effectiveness of these antivirals can be compromised by viral evolution. Mutations, such as H274Y in the NA gene, have been shown to confer resistance to oseltamivir, reducing the drug’s efficacy [79, 80]. In cases of antiviral resistance or the appearance of symptoms after treatment with oseltamivir, as an interim measure the CDC has recommended the use of baloxavir [81], which has been reported as effective in treating infections with H5N6 virus [82].

Fortunately, no evidence for the presence of this mutation or any other mutations conferring antiviral resistance has been found in infected dairy cattle or in the infected farm workers.

As avian influenza (H5N1) cases increase among cattle in the United States, global efforts are ramping up to develop and distribute vaccines to prevent potential human transmission. Vaccines are a critical component of pandemic preparedness, and as of July 2024, both Europe and the U.S. have approved vaccines to protect humans against the H5N1 influenza virus [83–86].

In 2020, the FDA approved Audenz, an adjuvanted monovalent vaccine developed by Seqirus, intended for adults to prevent disease caused by the H5N1 influenza virus subtype [87]. In 2013, Glaxo-Smith Kline received approval for its adjuvanted pandemic Influenza A (H5N1) Virus Monovalent Vaccine, also known as Q-Pan H5N1 influenza vaccine, for immunization of adults 18 and older [88], although its efficiency against clade 2.3.4.4b is low [89].

In the European Union, the European Medicines Agency (EMA) has recommended several vaccines against H5N1 virus, including Celldemic and Incellipan, both developed by Seqirus Netherlands B.V. [90]. These vaccines are designed for active immunization against avian influenza and are part of the EU’s pandemic preparedness strategy.

Recent actions include the European Commission securing 700,000 doses of an H5 strain vaccine, with the option to acquire 40 million more [86], and Finland starting to vaccinate high-risk workers. The U.S. Department of Health and Human Services (HHS) has moved forward with plans to produce 4.8 million doses of the H5N1 avian influenza vaccine to enhance pandemic preparedness [91]. Official estimations indicate that over 100 million doses could be distributed within three to four months. However, since two doses are required per person, this supply would be sufficient for only 50 million people [92], a very low threshold compared to the U.S. population of over 340 million. In clear contrast, by mid-2021, approximately three billion doses of COVID-19 vaccines had been administered globally, helping stop the spreading of the disease, but even that comparatively high number was insufficient due to significant inequity in the distribution, which focused mostly on high-income countries [93].

Additionally, researchers are developing mRNA vaccines, which can be produced more rapidly and updated quickly to address new strains. The U.S. Department of Health and Human Services has invested $176 million in Moderna for the development of an mRNA-based H5 vaccine. Ensuring fair global distribution is essential, with the Coalition for Epidemic Preparedness Innovations (CEPI) striving to make sure low- and middle-income countries are not neglected.

Vaccinating cattle to reduce transmission is also being explored, though there are challenges in effectively targeting the virus in cows. Research teams are in the early stages of developing both conventional and mRNA vaccines for livestock. Despite these preparedness efforts, the available vaccine supply is very scarce in the context of a worldwide health emergency.

## Pandemic preparedness requires a One Health approach

Wastewater surveillance by the CDC has detected the presence of influenza A virus in several states and cities across the U.S. [94]. A virome sequencing study identified HPAI H5N1 clade 2.3.4.4b in wastewater from nine of the ten monitored cities in Texas, U.S. [95]. Although the variant analysis in this study indicates an avian or bovine origin, other potential sources, especially humans, cannot be ruled out; additionally, the CDC surveillance methods are not as specific as those used in this study, so it is not possible to identify subtypes at the national level.

The uncertainty surrounding the types of influenza viruses present in wastewater further complicates public health responses and underscores the need for ongoing monitoring and analysis.

Current measures focus on containment and mitigation, but a proactive approach is urgently needed. Strengthening surveillance systems, increasing testing capacity, investing in research for vaccines and treatments, and fostering international cooperation to exchange data and stockpile vaccines are critical steps. In the longer term, addressing underlying factors such as intensive farming practices and wildlife trade, which create environments conducive to viral mutations, is crucial in preventing the next pandemic.

As a whole, the outbreak in dairy cattle underscores the interconnectedness of human, animal, and environmental health. The “One Health” approach, which emphasizes the collaboration of multiple sectors to achieve optimal health outcomes, is particularly relevant in this context [96, 97]. Efforts to prevent and control HPAI H5N1 must involve a comprehensive approach that considers the health of all species and their environments.

## Conclusion

The detection of the HPAI H5N1 virus in dairy cattle and the recent infections in humans and various mammalian species signal an urgent need for immediate and coordinated action. This evolving situation underscores the potential for the H5N1 virus to adapt and pose significant public health risks. The rapid evolution of the virus, coupled with its expanding host range, highlights the necessity for robust surveillance systems, increasing testing capacity, continuous research, and adaptive strategies for antiviral treatments and vaccines. Moreover, the potential for the virus to reassort and create new strains in both mixed-species environments and mixing vessels further complicates these efforts, necessitating a flexible and dynamic approach to vaccine and antiviral development.

Given the profound implications for global health, a proactive One Health approach is urgent. This approach must involve collaboration across human, animal, and environmental health sectors to implement comprehensive biosecurity measures, enhance surveillance, and ensure rapid response to outbreaks. Immediate actions should include increased surveillance and testing capacity, increased funding for research, stockpiling and development of next-generation vaccines and antivirals, and the establishment of a global network for real-time data sharing and coordination.

Are we cultivating the perfect storm for a human avian influenza pandemic? The likely answer is not “if” but “when”. Our ability to respond effectively and proactively to these emerging threats will determine our preparedness for the next global health crisis. The current HPAI H5N1 outbreak serves as a stark reminder of the need for global preparedness and action. The scientific community, policymakers, and international health organizations must collaborate to address this threat, preventing HPAI H5N1 from becoming a pandemic and protecting public health worldwide.

## Data Availability

Not applicable.

## References

[1] Subbarao K, Klimov A, Katz J, Regnery HL, Lim W, Hall H, Perdue M, Swayne D, Bender C, Huang J, Hemphill M, Rowe T, Shaw M, Xu X, Fukuda K, Cox N. Characterization of an avian influenza A (H5N1) virus isolated from a child with a fatal respiratory illness. Science. 1998;279(5349):393–6. 10.1126/SCIENCE.279.5349.393. 9430591 10.1126/science.279.5349.393

[2] Swayne D, Suarez D. Highly pathogenic avian influenza. Revue scientifique et technique-office international des epizooties. 2000;19(2):463–75. 10.20506/rst.19.2.1230 10935274

[3] Pereira H, Tumová B, Law V. Avian influenza A viruses. Bull World Health Org. 1965;32(6):855. 5294310 PMC2555286

[4] Stephenson I, Democratis J. Influenza: current threat from avian influenza. Br Med Bull. 2005;75–76:63–80. 10.1093/BMB/LDH063. 16651383 10.1093/bmb/ldh063

[5] Martin V, Pfeiffer D, Zhou X, Xiao X, Prosser D, Guo F, Gilbert M. Spatial distribution and risk factors of highly pathogenic avian influenza (HPAI) H5N1 in China. PLoS Pathogens. 2011. 10.1371/journal.ppat.1001308. 21408202 10.1371/journal.ppat.1001308 PMC3048366

[6] Centers for Disease Control and Prevention: Technical Report: June 2024 Highly Pathogenic Avian Influenza A(H5N1) Viruses. 2024. https://www.cdc.gov/bird-flu/php/technical-report/h5n1-06052024.html. Accessed 18 July 2024.

[7] Patel RB, Mathur MB, Gould M, Uyeki T, Bhattacharya J, Xiao Y, Khazeni N. Demographic and clinical predictors of mortality from highly pathogenic avian influenza A (H5N1) virus infection: CART analysis of international cases. PLoS ONE. 2014. 10.1371/journal.pone.0091630. 24667532 10.1371/journal.pone.0091630 PMC3965392

[8] Fasanmi OG, Laleye AT, Fasina FO. Systematic review and meta-analyses of cases and deaths associated with highly pathogenic avian influenza H5N1 in humans and poultry. CABI Rev. 2016;2016:1–12.

[9] Wu Z, Zhang Y, Zhao N, Yu Z, Pan H, Chan T, Zhang Z, Liu S. Comparative epidemiology of human fatal infections with novel, high (H5N6 and H5N1) and low (H7N9 and H9N2) pathogenicity avian influenza A viruses. Int J Environ Res Public Health. 2017. 10.3390/ijerph14030263. 28273867 10.3390/ijerph14030263 PMC5369099

[10] Yoo D-S, Kang S-I, Lee Y, Lee E-K, Kim W-Y, Lee Y-J. Bridging the local persistence and long-range dispersal of highly pathogenic avian influenza virus (HPAIv): a case study of HPAIv-infected sedentary and migratory wildfowls inhabiting infected premises. Viruses. 2022. 10.3390/v14010116. 35062320 10.3390/v14010116 PMC8780574

[11] Kapczynski, D.: Control strategies for 2.3.4.4 highly pathogenic avian influenza virus - from vaccines to host disease resistance. The First International Avian Influenza Summit. The University of Arkansas. October 16–17, 2023 (2023) 10.51585/gtop.2023.1.0020

[12] World Health Organization: Assessment of Risk Associated with Recent Influenza A(H5N1) Clade 2.3.4.4b Viruses. 2024. https://www.who.int/publications/m/item/assessment-of-risk-associated-with-recent-influenza-a. Accessed 26 Aug 2024.

[13] Adlhoch C, Fusaro A, Gonzáles J, Kuiken T, Marangon S, Niqueux E, Staubach C, Terregino C, Guajardo IPM, Chuzhakina K, Baldinelli F. Avian influenza overview June–September 2022. EFSA J. 2022. 10.2903/j.efsa.2022.7597. 36247870 10.2903/j.efsa.2022.7597 PMC9552036

[14] Rubio, A.G.: HPAI H5N1 outbreak in Chile, 2022-2023. The First International Avian Influenza Summit. The University of Arkansas. October 16–17, 2023. 2023. 10.51585/gtop.2023.1.0015

[15] Abbasi J. Bird flu outbreak in dairy cows is widespread, raising public health concerns. JAMA. 2024;331(21):1789–91. 10.1001/jama.2024.8886. 38718040 10.1001/jama.2024.8886

[16] United States Department of Agriculture: HPAI detections in livestock. 2024. https://www.aphis.usda.gov/livestock-poultry-disease/avian/avian-influenza/hpai-detections/livestock. Accessed 29 Aug 2024.

[17] USDA: USDA and HHS announce new actions to reduce the impact and spread of H5N1. 2024. https://www.usda.gov/media/press-releases/2024/05/10/usda-hhs-announce-new-actions-reduce-impact-and-spread-h5n1. Accessed 23 May 2024.

[18] Association, A.V.M.: Federal government allocates $200M to stop the spread of H5N1 among dairy cows. 2024. https://www.avma.org/news/200m-federal-government-aims-stop-spread-h5n1-among-dairy-cows. Accessed 23 May 2024.

[19] Spackman E, Jones DR, McCoig AM, Colonius TJ, Goraichuk IV, Suarez DL. Characterization of highly pathogenic avian influenza virus in retail dairy products in the US. medRxiv. 2024. 10.1101/2024.05.21.24307706 10.1128/jvi.00881-24 PMC11264905 38958444

[20] U.S. Food and Drug Administration: Updates on Highly Pathogenic Avian Influenza (HPAI). 2023. https://www.fda.gov/food/alerts-advisories-safety-information/updates-highly-pathogenic-avian-influenza-hpai. Accessed 16 May 2024.

[21] PBS NewsHour: Raw Milk Sales Spike Despite CDC’s Warnings of Risk Associated With Bird Flu. 2024. https://www.pbs.org/newshour/health/raw-milk-sales-spike-despite-cdcs-warnings-of-risk-associated-with-bird-flu. Accessed 27 May 2024.

[22] National Institutes of Health: Infectious H5N1 influenza virus in raw milk rapidly declines with heat treatment. 2024. https://www.nih.gov/news-events/news-releases/infectious-h5n1-influenza-virus-raw-milk-rapidly-declines-heat-treatment. Accessed 18 July 2024.

[23] U.S. Food and Drug Administration: questions and answers regarding milk safety during highly pathogenic avian influenza (HPAI) outbreaks. 2024. https://www.fda.gov/food/milk-guidance-documents-regulatory-information/questions-and-answers-regarding-milk-safety-during-highly-pathogenic-avian-influenza-hpai-outbreaks. Accessed 23 May 2024.

[24] Guan L, Eisfeld AJ, Pattinson D, Gu C, Biswas A, Maemura T, Trifkovic S, Babujee L, Presler R, Dahn R, Halfmann PJ, Barnhardt T, Neumann G, Thompson A, Swinford AK, Dimitrov KM, Poulsen K, Kawaoka Y. Cow’s milk containing avian influenza A(H5N1) virus - heat inactivation and infectivity in mice. N Engl J Med. 2024;391(1):87–90. 10.1056/NEJMc2405495. 38785313 10.1056/NEJMc2405495 PMC12809964

[25] Maxmen A, Allen A. Lack of bird flu tests could hide pandemic warning signs. 2024. https://www.scientificamerican.com/article/lack-of-bird-flu-tests-could-hide-pandemic-warning-signs/. Accessed 19 July 2024.

[26] Maxmen A. U.S. is ’flying blind’ with bird flu, repeating mistakes of COVID, health experts say. 2024. https://www.npr.org/sections/shots-health-news/2024/06/24/nx-s1-5015736/bird-flu-outbreak-testing-pandemic-preparedness. Accessed 19 July 2024.

[27] Apoorva Mandavilli LQ, Anthes E. How poor tracking of bird flu leaves dairy workers at risk. 2024. https://www.nytimes.com/2024/05/09/health/bird-flu-diaries-farmworkers.html. Accessed 23 May 2024.

[28] STAT News: CDC Looks to expand capacity to test for H5N1 bird flu in people. 2024. https://www.statnews.com/2024/07/02/cdc-looks-to-expand-capacity-to-test-for-h5n1-bird-flu-in-people/. Accessed 19 July 2024.

[29] Centers for Disease Control and Prevention: Monitoring for H5 Bird Flu. 2024. https://www.cdc.gov/bird-flu/h5-monitoring/index.html. Accessed 19 July 2024.

[30] Sun L, Roubein R, Diamond D. Bird flu virus found in grocery milk as officials say supply still safe. 2024. https://www.washingtonpost.com/health/2024/04/23/bird-flu-virus-milk/. Accessed 5 June 2024.

[31] Cohen J, Enserink M. Bird flu appears entrenched in US dairy herds. Science. 2024;384(6695):493–4. 38696559 10.1126/science.adq1771

[32] Reardon S. Bird flu in us cows: where will it end? Nature. 2024;629:515–6. 10.1038/d41586-024-01333-9. 38714908 10.1038/d41586-024-01333-9

[33] Centers for Disease Control and Prevention: CDC Reports Fourth Human Case of H5 Bird Flu in the U.S. 2024. https://www.cdc.gov/media/releases/2024/p-0703-4th-human-case-h5.html. Accessed 19 July 2024.

[34] Mallapaty S. Bird flu virus has been spreading among US cows for months, RNA reveals. Nature. 2024;629:490–1. 10.1038/d41586-024-01256-5. 10.1038/d41586-024-01256-5 38678111

[35] Centers for Disease Control and Prevention: 2023-2024 Bird Flu Update. 2024. https://www.cdc.gov/flu/avianflu/spotlights/2023-2024/bird-flu-update_05242024.html. Accessed 27 May 2024.

[36] Nguyen T-Q, Hutter C, Markin A, Thomas M, Lantz K, Killian ML, Janzen GM, Vijendran S, Wagle S, Inderski B, Magstadt DR, Li G, Diel DG, Frye EA., Dimitrov KM, Swinford AK, Thompson AC, Snevik KR, Suarez DL, Spackman E, Lakin SM, Ahola SC, Johnson KR, Baker AL, Robbe-Austerman S, Torchetti MK, Anderson TK. Emergence and interstate spread of highly pathogenic avian influenza A(H5N1) in dairy cattle. bioRxiv; 2024. 10.1101/2024.05.01.591751.39677809

[37] Uyeki TM, Milton S, Hamid CA, Webb CR, Presley SM, Shetty V, Rollo SN, Martinez DL, Rai S, Gonzales ER, Kniss KL, Jang Y, Frederick JC, Cruz JADL, Liddell J, Di H, Kirby MK, Barnes JR, Davis CT. Highly pathogenic avian influenza A(H5N1) virus infection in a dairy farm worker. N Engl J Med. 2024;390(21):2028–9. 10.1056/NEJMc2405371. 38700506 10.1056/NEJMc2405371

[38] Danzy S, Studdard LR, Manicassamy B, Solórzano A, Marshall N, García-Sastre A, Steel J, Lowen AC. Mutations to PB2 and NP proteins of an avian influenza virus combine to confer efficient growth in primary human respiratory cells. J Virol. 2014;88:13436–46. 10.1128/JVI.01093-14. 25210184 10.1128/JVI.01093-14 PMC4249088

[39] Zhang H, Li X, Guo J, Li L, Chang C, Li Y, Bian C, Xu K, Chen H, Sun B. The PB2 E627K mutation contributes to the high polymerase activity and enhanced replication of H7N9 influenza virus. J General Virol. 2014;95:779–86. 10.1099/vir.0.061721-0. 10.1099/vir.0.061721-0 24394699

[40] Zhu W, Li L, Yan Z, Gan T, Li L, Chen R, Chen R, Zheng Z, Hong W, Wang J, Smith DK, Guan Y, Zhu H, Shu Y. Dual E627K and D701N mutations in the PB2 protein of A(H7N9) influenza virus increased its virulence in mammalian models. Sci Rep. 2015. 10.1038/srep14170. 26391278 10.1038/srep14170 PMC4585756

[41] Centers for Disease Control and Prevention: H5N1 Technical Update - May 24, 2024. 2024. https://www.cdc.gov/flu/avianflu/spotlights/2023-2024/h5n1-technical-update-may-24-2024.html. Accessed 27 May 2024.

[42] Centers for Disease Control and Prevention: CDC confirms second human H5 bird flu case in Michigan; Third Case Tied to Dairy Outbreak. 2024. https://www.cdc.gov/media/releases/2024/p0530-h5-human-case-michigan.html. Accessed 5 June 2024.

[43] Restori KH, Septer KM, Field CJ, Patel DR, VanInsberghe D, Raghunathan V, Lowen AC, Sutton TC. Risk assessment of a highly pathogenic H5N1 influenza virus from mink. Nat Commun. 2024;15:4112. 10.1038/s41467-024-48475-y. 38750016 10.1038/s41467-024-48475-y PMC11096306

[44] Bussey KA, Bousse T, Desmet EA, Kim B, Takimoto T. PB2 residue 271 plays a key role in enhanced polymerase activity of influenza A viruses in mammalian host cells. J Virol. 2010;84:4395–406. 10.1128/JVI.02642-09. 20181719 10.1128/JVI.02642-09 PMC2863787

[45] Agüero M, Monne I, Sánchez A, Zecchin B, Fusaro A, Ruano MJ, Valle Arrojo M, Fernández-Antonio R, Souto AM, Tordable P. Highly pathogenic avian influenza A (H5N1) virus infection in farmed minks, Spain, October 2022. Eurosurveillance. 2023;28(3):2300001. 36695488 10.2807/1560-7917.ES.2023.28.3.2300001 PMC9853945

[46] Feng Z, Zhu W, Zhou L, Chen Y, Li X, Gao R, Liu J, Wang D, Shu Y. The substitution of T271A in PB2 protein could enhance the infectivity and pathogenicity of eurasian avian-like H1N1 swine influenza viruses in mice. 2020. 10.21203/rs.3.rs-27732/v1https://www.researchsquare.com/

[47] Ito T, Couceiro J, Kelm S, Baum L, Krauss S, Castrucci M, Donatelli I, Kida H, Paulson J, Webster R, Webster R, Kawaoka Y, Kawaoka Y. Molecular basis for the generation in pigs of influenza A viruses with pandemic potential. J Virol. 1998;72:7367–73. 10.1128/JVI.72.9.7367-7373.1998. 9696833 10.1128/jvi.72.9.7367-7373.1998 PMC109961

[48] Castillo A, Fasce R, Parra B, Andrade W, Covarrubias P, Hueche A, Campano C, Tambley C, Rojas M, Araya M. The first case of human infection with H5N1 avian influenza A virus in Chile. J Travel Med. 2023;30(5):083. 10.1093/jtm/taad083 PMC10481412 37310882

[49] Bruno A, Alfaro-Núñez A, Mora D, Armas R, Olmedo M, Garcés J, Garcia-Bereguiain MA. First case of human infection with highly pathogenic H5 avian influenza A virus in South America: a new zoonotic pandemic threat for 2023? J Travel Med. 2023;30(5):032. 10.1093/jtm/taad032 PMC10481407 36881656

[50] Czudai-Matwich V, Otte A, Matrosovich M, Gabriel G, Klenk H. PB2 mutations D701N and S714R promote adaptation of an influenza H5N1 virus to a mammalian host. J Virol. 2014;88:8735–42. 10.1128/JVI.00422-14. 24899203 10.1128/JVI.00422-14 PMC4136279

[51] Li Q, Wang X, Sun Z, Hu J, Gao Z, Hao X, Li J, Liu H, Wang X, Gu M, Xu X, Liu X, Liu X. Adaptive mutations in PB2 gene contribute to the high virulence of a natural reassortant H5N2 avian influenza virus in mice. Virus Res. 2015;210:255–63. 10.1016/j.virusres.2015.08.017. 26315686 10.1016/j.virusres.2015.08.017

[52] Steel J, Lowen AC, Mubareka S, Palese P. Transmission of influenza virus in a mammalian host is increased by PB2 amino acids 627K or 627E/701N. PLoS Pathogens. 2009. 10.1371/journal.ppat.1000252. 19119420 10.1371/journal.ppat.1000252 PMC2603332

[53] Zhou, B., Pearce, M.B., Li, Y., Wang, J., Mason, R., Tumpey, T., Wentworth, D. Asparagine substitution at PB2 residue 701 enhances the replication, pathogenicity, and transmission of the pandemic H1N1 influenza A virus. PLoS ONE. 2009. 10.1371/journal.pone.0067616. 23799150 10.1371/journal.pone.0067616 PMC3683066

[54] Le QM, Ito M, Muramoto Y, Hoang PVM, Vuong CD, Sakai-Tagawa Y, Kiso M, Ozawa M, Takano R, Kawaoka Y. Pathogenicity of highly pathogenic avian H5N1 influenza A viruses isolated from humans between 2003 and 2008 in northern Vietnam. J General Virol. 2010;91(10):2485–90. 10.1099/vir.0.021659-0 PMC3052597 20592108

[55] Zhu W, Li X, Dong J, Bo H, Liu J, Yang J, Zhang Y, Wei H, Huang W, Zhao X. Epidemiologic, clinical, and genetic characteristics of human infections with influenza A (H5N6) viruses. China Emerg Infect Dis. 2022;28(7):1332. 35476714 10.3201/eid2807.212482 PMC9239879

[56] Carvalho Araujo A., Cho AY, Silva LMN, Corrêa TC, Souza GC, Albuquerque AS, Macagnan E, Kolesnikvoas CK, Meurer R, Vieira JV. Mortality in sea lions is associated with the introduction of the H5N1 clade 2.3.4.4b virus in Brazil October 2023: whole genome sequencing and phylogenetic analysis. BMC Vet Res. 2024;20(1):285. 38956597 10.1186/s12917-024-04137-1 PMC11221036

[57] Leguia M, Garcia-Glaessner A, Muñoz-Saavedra B, Juarez D, Barrera P, Calvo-Mac C, Jara J, Silva W, Ploog K, Amaro L. Highly pathogenic avian influenza A (H5N1) in marine mammals and seabirds in Peru. Nat Commun. 2023;14(1):5489. 37679333 10.1038/s41467-023-41182-0 PMC10484921

[58] Plaza P, Gamarra-Toledo V, Euguí JR, Lambertucci S. Recent changes in patterns of mammal infection with highly pathogenic avian influenza A(H5N1) virus worldwide. Emerg Infect Dis J. 2024;30(3):444. 10.3201/eid3003.231098. 10.3201/eid3003.231098 PMC10902543 38407173

[59] Hu X, Saxena A, Magstadt DR, Gauger PC, Burrough E, Zhang J, Siepker C, Mainenti M, Gorden PJ, Plummer P, Li G. Highly pathogenic avian influenza A (H5N1) clade 2.3.4.4b virus detected in dairy cattle. bioRxiv. 2024. 10.1101/2024.04.16.588916.39677822

[60] Yamada S, Suzuki Y, Suzuki T, Le MQ, Nidom CA, Sakai-Tagawa Y, Muramoto Y, Ito M, Kiso M, Horimoto T. Haemagglutinin mutations responsible for the binding of H5N1 influenza A viruses to human-type receptors. Nature. 2006;444(7117):378–82. 17108965 10.1038/nature05264

[61] Gao Y, Zhang Y, Shinya K, Deng G, Jiang Y, Li Z, Guan Y, Tian G, Li Y, Shi J. Identification of amino acids in HA and PB2 critical for the transmission of H5N1 avian influenza viruses in a mammalian host. PLoS Pathogens. 2009;5(12):1000709. 10.1371/journal.ppat.1000709 PMC2791199 20041223

[62] Kristensen C, Jensen HE, Trebbien R, Webby RJ, Larsen LE. The avian and human influenza A virus receptors sialic acid (SA)-alpha2, 3 and SA-alpha2, 6 are widely expressed in the bovine mammary gland. bioRxiv. 2024. 10.1101/2024.05.03.592326.37745562

[63] Oxford J, Lambkin R, Sefton A, Daniels R, Elliot A, Brown R, Gill D. A hypothesis: the conjunction of soldiers, gas, pigs, ducks, geese and horses in northern France during the Great War provided the conditions for the emergence of the “Spanish” influenza pandemic of 1918–1919. Vaccine. 2005;23(7):940–5. 10.1016/J.VACCINE.2004.06.035. 15603896 10.1016/j.vaccine.2004.06.035

[64] Mena I, Nelson MI, Quezada-Monroy F, Dutta J, Cortes-Fernández R, Lara-Puente JH, Castro-Peralta F, Cunha LF, Trovão NS, Lozano-Dubernard B. Origins of the 2009 H1N1 influenza pandemic in swine in Mexico. elife. 2016;5:16777. 10.7554/eLife.16777 PMC4957980 27350259

[65] Torremorell M, Allerson M, Corzo C, Diaz A, Gramer M. Transmission of influenza A virus in pigs. Transb Emerg Dis. 2012;59(Suppl1):68–84. 10.1111/j.1865-1682.2011.01300.x. 10.1111/j.1865-1682.2011.01300.x 22226050

[66] Burrough ER, Magstadt DR, Petersen B, Timmermans SJ, Gauger PC, Zhang J, Siepker C, Mainenti M, Li G, Thompson AC, Gorden PJ, Plummer PJ, Main R. Highly pathogenic avian influenza A(H5N1) clade 2.3.4.4b virus infection in domestic dairy cattle and cats, United States, 2024. Emerg Infect Dis. 2024;30(7):1335. 38683888 10.3201/eid3007.240508 PMC11210653

[67] Schnirring, L.: Analysis of cow, cat H5N1 avian flu samples raises concerns about spread to other animals. Center for Infectious Disease Research and Policy, University of Minnesota (2024). https://www.cidrap.umn.edu/avian-influenza-bird-flu/analysis-cow-cat-h5n1-avian-flu-samples-raises-concerns-about-spread-other

[68] Wang G, Zhang J, Li W, Xin G, Su Y, Gao Y, Zhang H, Lin G-M, Jiao X, Li K. Apoptosis and proinflammatory cytokine responses of primary mouse microglia and astrocytes induced by human H1N1 and avian H5N1 influenza viruses. Cell Mol Immunol. 2008;5:113–20. 10.1038/cmi.2008.14. 18445341 10.1038/cmi.2008.14 PMC4651245

[69] Yamada M, Bingham J, Payne JS, Rookes J, Lowther S, Haining J, Robinson R, Johnson D, Middleton D. Multiple routes of invasion of wild-type clade 1 highly pathogenic avian influenza H5N1 virus into the central nervous system (CNS) after intranasal exposure in ferrets. Acta Neuropathologica. 2012;124:505–16. 10.1007/s00401-012-1010-8. 22763823 10.1007/s00401-012-1010-8

[70] Rijks J, Hesselink H, Lollinga P, Wesselman R, Prins P, Weesendorp E, Engelsma M, Heutink R, Harders F, Kik M, Rozendaal H, Kerkhof H, Beerens N. Highly pathogenic avian influenza A(H5N1) virus in wild red foxes, the Netherlands, 2021. Emerg Infect Dis. 2021;27:2960–2. 10.3201/eid2711.211281. 34670656 10.3201/eid2711.211281 PMC8544991

[71] Siegers JY, Ferreri L, Eggink D, Kroeze EV, Velthuis AJT, Bildt MVD, Leijten L, Run P, Meulder D, Bestebroer T, Richard M, Kuiken T, Lowen AC, Herfst S, Riel D. Evolution of highly pathogenic H5N1 influenza A virus in the central nervous system of ferrets. PLOS Pathogens. 2023. 10.1371/journal.ppat.1011214. 36897923 10.1371/journal.ppat.1011214 PMC10032531

[72] Tanaka H, Park C-H, Ninomiya A, Ozaki H, Takada A, Umemura T, Kida H. Neurotropism of the 1997 Hong Kong H5N1 influenza virus in mice. Vet Microbiol. 2003;95(1–2):1–13. 10.1016/S0378-1135(03)00132-9. 12860072 10.1016/s0378-1135(03)00132-9

[73] Lipatov AS, Krauss S, Guan Y, Peiris M, Rehg JE, Perez DR, Webster RG. Neurovirulence in mice of H5N1 influenza virus genotypes isolated from Hong Kong poultry in 2001. J Virolgy. 2003;77(6):3816–23. 10.1128/JVI.77.6.3816-3823.2003 PMC149508 12610156

[74] Dankar S, Miranda E, Forbes N, Pelchat M, Tavassoli A, Selman M, Ping J, Jia J, Brown E. Influenza A/Hong Kong/156/1997(H5N1) virus NS1 gene mutations F103L and M106I both increase IFN antagonism, virulence and cytoplasmic localization but differ in binding to RIG-I and CPSF30. Virol J. 2013;10:243–243. 10.1186/1743-422X-10-243. 23886034 10.1186/1743-422X-10-243 PMC3733596

[75] Chen L, Wang C, Luo J, Li M, Liu H, Zhao N, Huang J, Zhu X, Ma G, Yuan G, He H. Amino acid substitution K470R in the nucleoprotein increases the virulence of H5N1 influenza A virus in mammals. Front Microbiol. 2017. 10.3389/fmicb.2017.01308. 28744280 10.3389/fmicb.2017.01308 PMC5504190

[76] Ilyushina N, Seiler J, Rehg J, Webster R, Govorkova E. Effect of neuraminidase inhibitor-resistant mutations on pathogenicity of clade 2.2 A/Turkey/15/06 (H5N1) influenza virus in ferrets. PLoS Pathogens. 2010. 10.1371/journal.ppat.1000933. 20523902 10.1371/journal.ppat.1000933 PMC2877746

[77] Mitrasinovic P. Advances in the structure-based design of the influenza A neuraminidase inhibitors. Curr Drug Targets. 2010;11(3):315–26. 10.2174/138945010790711932. 20210756 10.2174/138945010790711932

[78] Russell R, Haire L, Stevens D, Collins P, Lin YP, Blackburn G, Hay A, Gamblin S, Skehel J. The structure of H5N1 avian influenza neuraminidase suggests new opportunities for drug design. Nature. 2006;443:45–9. 10.1038/nature05114. 16915235 10.1038/nature05114

[79] Legoff J, Rousset D, Abou-Jaoude G, Scemla A, Ribaud P, Mercier-Delarue S, Caro V, Enouf V, Simon F, Molina J, Werf S. I223R mutation in influenza A(H1N1)pdm09 neuraminidase confers reduced susceptibility to Oseltamivir and Zanamivir and enhanced resistance with H275Y. PLoS ONE. 2012. 10.1371/journal.pone.0037095. 22936969 10.1371/journal.pone.0037095 PMC3427316

[80] Abed Y, Nehmé B, Baz M, Boivin G. Activity of the neuraminidase inhibitor A-315675 against oseltamivir-resistant influenza neuraminidases of N1 and N2 subtypes. Antiviral Res. 2008;77(2):163–6. 10.1016/J.ANTIVIRAL.2007.08.008. 17919743 10.1016/j.antiviral.2007.08.008

[81] CDC: Interim Guidance on the Use of Antiviral Medications for Treatment of Human Infections with Novel Influenza A Viruses Associated with Severe Human Disease

[82] Guan W, Qu R, Shen L, Mai K, Pan W, Lin Z, Chen L, Dong J, Zhang J, Feng P. Baloxavir marboxil use for critical human infection of avian influenza A H5N6 virus. Med. 2024;5(1):32–41. 38070511 10.1016/j.medj.2023.11.001

[83] Oshansky CM, Zhou J, Gao Y, Schweinle JE, Biscardi K, DeBeauchamp J, Pavetto C, Wollish A, Webby RJ, Cioce V. Safety and immunogenicity of influenza A (H5N1) vaccine stored up to twelve years in the National Pre-Pandemic Influenza Vaccine Stockpile (NPIVS). Vaccine. 2019;37(3):435–43. 30553570 10.1016/j.vaccine.2018.11.069

[84] Versage E, Twuijver E, Jansen W, Theeuwes A, Sawlwin D, Hohenboken M. Analyses of safety profile and homologous antibody responses to a mammalian cell-based, MF59-adjuvanted, A/H5N1, pandemic influenza vaccine across four phase II/III clinical trials in healthy children, adults, and older adults. Vaccines. 2021;9(12):1468. 34960214 10.3390/vaccines9121468 PMC8704792

[85] U.S. Food and Drug Administration: Vaccines Licensed for Use in the United States. Accessed: 2024-08-01 (2024). https://www.fda.gov/vaccines-blood-biologics/vaccines/vaccines-licensed-use-united-states

[86] European Commission: Commission secures 700,000 doses of H5 strain vaccine with option for 40 million more. 2024. https://ec.europa.eu/commission/presscorner/detail/en/IP_24_3168. Accessed 1 Aug 2024.

[87] U.S. Food and Drug Administration: AUDENZ. 2024. https://www.fda.gov/vaccines-blood-biologics/audenz. Accessed 26 May 2024.

[88] GlaxoSmithKline: H5N1 vaccine approved by the US FDA as pandemic influenza preparedness measure. 2020. https://www.gsk.com/en-gb/media/press-releases/h5n1-vaccine-approved-by-the-us-fda-as-pandemic-influenza-preparedness-measure/. Accessed 26 May 2024.

[89] Kim JH, Drame M, Puthanakit T, Chiu N-C, Supparatpinyo K, Huang L-M, Chiu C-H, Chen P-Y, Hwang K-P, Danier J. Immunogenicity and safety of AS03-adjuvanted H5N1 influenza vaccine in children 6–35 months of age: results from a phase 2, randomized, observer-blind, multicenter, dose-ranging study. Pediatric Infect Dis J. 2021;40(9):333–9. 10.1097/INF.0000000000003247 PMC8357047 34285165

[90] European Medicines Agency: Meeting highlights from the Committee for Medicinal Products for Human Use (CHMP) 19–22 February 2024. 2024.https://www.ema.europa.eu/en/news/meeting-highlights-committee-medicinal-products-human-use-chmp-19-22-february-2024. Accessed 26 May 2024.

[91] Schnirring L. HHS advances plan to produce 4.8 million H5N1 vaccine doses. CIDRAP (2024). https://www.cidrap.umn.edu/avian-influenza-bird-flu/hhs-advances-plan-produce-48-million-h5n1-vaccine-doses

[92] Radcliffe, S.: Bird Flu: U.S. Could Produce and Ship 100 Million Vaccine Doses Within Months. 2024. https://www.healthline.com/health-news/bird-flu-u-s-could-produce-and-ship-100-million-vaccine-doses-within-months

[93] Sharma K, Koirala A, Nicolopoulos K, Chiu C, Wood N, Britton P. Vaccines for COVID-19: where do we stand in 2021? Paediatric Respir Rev. 2021;39:22–31. 10.1016/j.prrv.2021.07.001. 10.1016/j.prrv.2021.07.001 PMC8274273 34362666

[94] Centers for Disease Control and Prevention: Influenza A Virus Wastewater Data. 2024. https://www.cdc.gov/nwss/wastewater-surveillance/Flu-A-data.html

[95] Tisza MJ, Hanson BM, Clark JR, Wang L, Payne K, Ross MC, Mena KD, Gitter A, Javornik-Cregeen SJ, Cormier J, Avadhanula V, Terwilliger A, Balliew J, Wu F, Rios J, Deegan J, Piedra PA, Petrosino JF, Boerwinkle E, Maresso AW. Virome sequencing identifies H5N1 avian influenza in wastewater from nine cities. medRxiv. 2024. 10.1101/2024.05.10.24307179.39040200

[96] Monath TP. Vaccines against diseases transmitted from animals to humans: a one health paradigm. Vaccine. 2013;31(46):5321–38. 24060567 10.1016/j.vaccine.2013.09.029 PMC7130581

[97] Degeling C, Johnson J, Kerridge I, Wilson A, Ward M, Stewart C, Gilbert G. Implementing a one health approach to emerging infectious disease: reflections on the socio-political, ethical and legal dimensions. BMC Public Health. 2015;15:1–11. 26715066 10.1186/s12889-015-2617-1 PMC4696140

